# Preclinical evaluation of safety and immunogenicity of a primary series intranasal COVID-19 vaccine candidate (BBV154) and humoral immunogenicity evaluation of a heterologous prime-boost strategy with COVAXIN (BBV152)

**DOI:** 10.3389/fimmu.2022.1063679

**Published:** 2022-12-08

**Authors:** Raju Sunagar, Sai D. Prasad, Raches Ella, Krishna Mohan Vadrevu

**Affiliations:** Bharat Biotech International Ltd, Hyderabad, India

**Keywords:** SARS-CoV-2, COVID-19 vaccine, BBV154, ChAd vaccine, intranasal vaccine, mucosal vaccine, heterologous, iNCOVACC

## Abstract

Most if not all vaccine candidates developed to combat COVID-19 due to SARS-CoV-2 infection are administered parenterally. As SARS-CoV-2 is transmitted through infectious respiratory fluids, vaccine-induced mucosal immunity could provide an important contribution to control this pandemic. ChAd-SARS-CoV-2-S (BBV154), a replication-defective chimpanzee adenovirus (ChAd)-vectored intranasal (IN) COVID-19 vaccine candidate, encodes a prefusion-stabilized version of the SARS-CoV-2 spike protein containing two proline substitutions in the S2 subunit. We performed preclinical evaluations of BBV154 in mice, rats, hamsters and rabbits. Repeated dose toxicity studies presented excellent safety profiles in terms of pathology and biochemical analysis. IN administration of BBV154 elicited robust mucosal and systemic humoral immune responses coupled with Th1 cell-mediated immune responses. BBV154 IN vaccination also elicited potent variant (omicron) cross neutralization antibodies. Assessment of anti-vector (ChAd36) neutralizing antibodies following repeated doses of BBV154 IN administration showed insignificant titers of ChAd36 neutralizing antibodies. However, the immune sera derived from the same animals displayed significantly higher levels of SARS-CoV-2 virus neutralization (p<0.003). We also evaluated the safety and immunogenicity of heterologous prime-boost vaccination with intramuscular (IM) COVAXIN-prime followed by BBV154 IN administration. COVAXIN priming followed by BBV154 IN-booster showed an acceptable reactogenicity profile comparable to the homologous COVAXIN/COVAXIN or BBV154/BBV154 vaccination model. Heterologous vaccination of COVAXIN-prime and BBV154 booster also elicited superior (p<0.005) and cross variant (omicron) protective immune responses (p<0.013) compared with the homologous COVAXIN/COVAXIN schedule. BBV154 has successfully completed both homologous and heterologous combination schedules of human phase 3 clinical trials and received the restricted emergency use approval (in those aged above 18 years) from the Drugs Controller General of India (DCGI).

## Introduction

The global spread of severe acute respiratory syndrome coronavirus 2 (SARS-CoV-2) that led to the COVID-19 pandemic has continued with waves of new outbreaks around the world. The initial pandemic made deployment of an effective vaccine a global health priority and there have been almost 200 vaccine candidates in various stages of development, with more than 172 entering human clinical trials (1). However, until now, the COVID-19 vaccine candidates that have received emergency use authorization by the FDA and other regulatory agencies have been based on intramuscular (IM) injections (1–3). These IM COVID-19 vaccines are designed to elicit robust systemic immunity, but induce limited mucosal immunity that is critical for blocking SARS-CoV-2 infection and transmission, leading to breakthrough infection in fully vaccinated individuals (4, 5). Maintaining control of the pandemic in the face of the increasing of numbers of mutated forms of SARS-CoV-2 variants that escape vaccine-induced immunity now requires further development of new vaccines and vaccination approaches.

Adenoviruses are considered to be a safe and well-studied platform for antigen delivery (6–8), and have been used successfully to develop a COVID-19 vaccine (9). The chimpanzee Ad-vectored (Y25, a simian Ad-23) vaccine candidate (10) encoding the spike protein of MERS-CoV is protective in mice (11) and camels (12), and was shown to be safe and immunogenic in humans (13). In rhesus macaques, intramuscular administration of the ChAdOx1 nCoV-19 vaccine encoding the spike (S) protein of SARS-CoV-2 provided protection against lung infection and pneumonia, but not against upper respiratory tract infection and virus shedding (4). In clinical trials, ChAdOx1 nCoV-19 was found to be safe, immunogenic and efficacious against SARS-CoV-2 pneumonia (14, 15) leading to WHO approval for its use against COVID-19. Recently, a novel chimpanzee Ad (simian Ad-36)-based SARS-CoV-2 vaccine (ChAd36-SARS-CoV-2-S) has been developed encoding a prefusion stabilized spike (S) protein with two proline substitutions in the S2 subunit (16). A single intranasal (IN) dose of ChAd36-SARS-CoV-2-S conferred superior protective immunity against SARS-CoV-2 challenge than one or two intramuscular (IM) doses of the same vaccine. Additionally, one IN dose of ChAd36-SARS-CoV-2-S prevented upper and lower respiratory tract infection and inflammation by SARS-CoV-2 in highly susceptible K18-hACE2 transgenic mice and Syrian hamsters (16, 17), with similar results in rhesus macaques (18).

GMP batches of ChAd36-SARS-CoV-2-S (BBV154) have now been manufactured and formulated as an IN COVID-19 vaccine candidate. Here, we report the safety and immunogenicity of BBV154 in laboratory animal models, i.e., mice, rats, hamsters and rabbits, following single or two dose IN administration, and heterologous prime-boost vaccination of BBV154 in combination with COVAXIN a whole virion inactivated SARS-CoV-2 vaccine (codenamed as BBV152) formulated with a toll-like receptor 7/8 agaonist molecule absorbed to alum developed by Bharat Biotech Int Ltd, India (19).

## Results

### Production of BBV154

**Figure 1:**
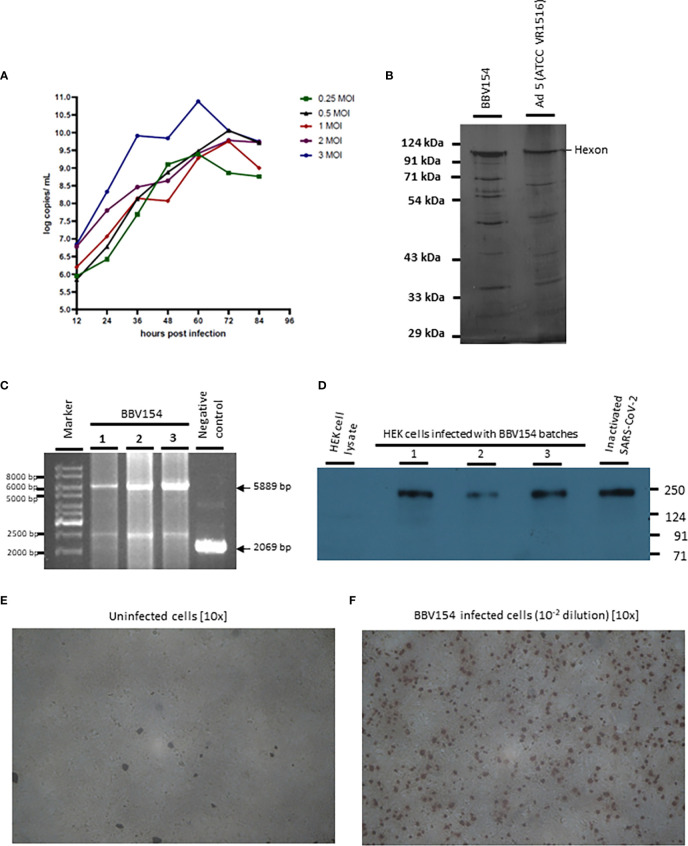
Characterization of BBV154 vaccine candidate. Growth kinetics of BBV154 in HEK cells was studied by infecting HEK cells with 0.25, 0.5, 1, 2 and 3 MOI of BBV154 virus. Samples were collected at 12 hours intervals and were subjected to qPCR analysis to estimate the genome copies of the virus **(A)**. SDS-PAGE and silver staining of BBV154 (drug product) and Ad5 reference reagent from ATCC **(B)**. DNA was extracted from three batches of BBV154 and primers flanking the expression cassette were used to detect its presence. Amplification of 5889 bp PCR product indicates the presence of the spike expression cassette. The DNA extracted from ChAd vector was used as negative control which amplified a 2069 bp PCR product which indicates the absence of the spike expression cassette. Marker: Gene ruler SM03111, Thermoscientific **(C)**. The HEK cells were infected with 5 MOI of three different batches of BBV154 or were left uninfected. Twenty-four hours post-infection the cells were harvested and lysed. The samples were subjected to western blotting with rabbit polyclonal antibody against RBD and anti-rabbit peroxidase conjugated antibody. Inactivated and purified SARS-CoV2 was used as positive control and Hfimmu.2022.1063679EK cell lysate was used as negative control **(D)**. The cells were either left uninfected **(E)** or infected with 10^-2^ dilution of BBV154 **(F)**. 48 hours post infection, the cells were fixed and probed with rabbit polyclonal S1 antibody followed by anti-rabbit IgG peroxidase. The peroxidase activity was detected by 3-Amino-9-Ethylcarbazole. BBV154 characterization assays were repeated at least three times using three batches of BBV154 drug substances.

The master and working virus banks of the BBV154 vaccine and the HEK cells were prepared in a GMP facility. Virus and cell banks were characterized for identity, safety and purity according to ICH guidelines. Infection of the HEK cells with the BBV154 produced a cytopathic effect (CPE) which included rounding of cells and lysis. Growth kinetics of the BBV154 in HEK cells was carried out with five different multiplicities of infection (MOI) ranging from 0.25 to 3. Based on the results of ChAd genome copies (**Figure 1A**) and infectious titer (data not shown) estimations of the growth kinetics sample, an optimum MOI of 1 and a harvest time of 60 hours post-infection were selected. The downstream purification cascade as mentioned in the Methods section was followed to obtain a purified and formulated BBV154 vaccine candidate (**Figure 1B**).

### Characterization of BBV154

The BBV154 vaccine candidate was tested for the integrity of the expression cassette by conventional PCR. The primers flanking the expression cassette were used to amplify an expected product of 5889 base pairs (bp) from three different batches. A PCR product of 2069 bp was obtained with the ChAd vector control (**Figure 1C**).

The spike expression by BBV154 was assessed by infection of HEK cells and the cell lysates were subjected to western blotting with antibody against the receptor binding domain (RBD) of spike protein (**Figure 1D**). The RBD antibody detected the full-length spike in the cell lysates derived from BBV154 infected cells (**Figure 1D**, lanes 2-4) and the spike protein size corresponds to that of the positive control (**Figure 1D**, lane 5). The spike expression of BBV154 was also demonstrated by immunocytochemistry, spike expression products being visualized as distinct spots (**Figures 1E, F**).

In order to test for the presence of replication competent adenovirus (RCA), BBV154 was passaged in A549 cells that do not have complement E1. As expected, CPE was not observed even after three consecutive passages indicating absence of RCA. To exclude the possibility of inhibition of the RCA in the BBV154 sample, BBV154 spiked with wild type Ad5 (1 or 10 TCID) displayed CPE with an amplification between 10^9^ to 10^10^ TCID_50_ (**Table 1**).

**Table 1:** Test for detection of RCA in the BBV154 sample.

Sample Details	Number of passages in A549 cells	TCID_50_ assay performed on	Virus titers after three passages (TCID_50_)
BBV154 sample alone*	3	A549 cells	No CPE
BBV154 sample spiked with 1 TCID of Ad5			10^10^
BBV154 sample spiked with 10 TCID of Ad5			10^10.35^
Ad5 alone 10 TCID			10^11^
BBV154 sample alone		HEK cells	No CPE

*Hexon copies: 10.58 log copies/mL; Spike copies: 10.18 log copies/mL; virus particle by A260: 2.82 x 10^12^ virus particle/mL.

### Safety evaluation of BBV154 formulation

An extensive safety evaluation for the vaccine candidate BBV154 formulation was performed as per the regulatory guidelines (20, 21). Safety of BBV154 was assessed by repeated dose toxicity studies conducted in both rodent (Swiss albino mice, BALB/c mice and Wistar rats) and non-rodent (New Zealand White rabbits) species, after the administration of N+1 dose regimen. No mortality, systemic toxicity or clinical signs was observed in any of the animal models throughout the tested experimental period. Body weight gain (**Figure S1**), food consumption and body temperature of the animals were within the normal range. Clinical pathology parameters such as hematology, clinical biochemistry, urinalysis, coagulation analysis and acute phase protein values did not indicate any variation in the vaccine-treated animals from placebo-treated animals even at the highest dose tested (5 x 10^11^ VP/animal). Further, the coagulation factors leading to thrombosis or thrombocytopenia, platelet count, prothrombin time and activated partial thromboplastin time (APTT) were well within the normal range and comparable with the concurrent control group and were unaffected by BBV 154 (**Tables S2**, **S3**). The skin around the nose did not show any local reactogenicity such as erythema, edema or eschar formation on gross observation. Absolute and relative (to body weight) organ weights were comparable to placebo animals. Further extensive histopathological evaluation, following Registry of Industrial Toxicology Animal-data (RITA) guidance, of three levels of nasal cavity (**Figure S2**) and related organs such as larynx, trachea, lungs or associated lymph nodes (**Figure S3**) did not reveal any abnormalities in microscopic observations.

### Immunogenicity of single dose vaccination of BBV154

**Figure 2:**
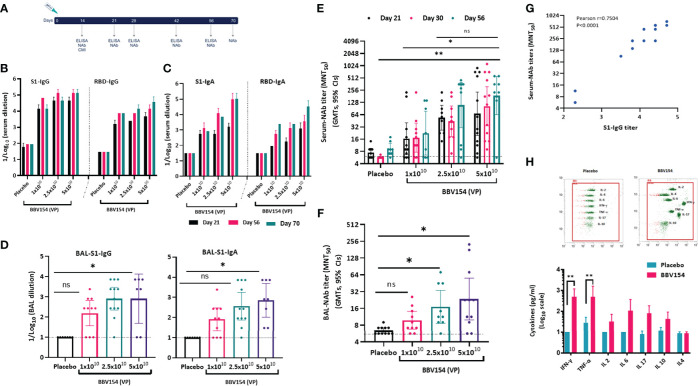
Candidate vaccine BBV154 elicits robust humoral and cell mediated immune responses against SARS-CoV-2. Schematic diagram of immunization regime mouse experiment **(A)**. Five to six-week-old BALB/c male and female mice were immunized with 50 μl of placebo or BBV154 *via* intranasal route either with one-tenth, one-quarter or half of a Human Single Dose (HSD). Antibody responses in sera of immunized mice at different time points were evaluated (B & C). SARS-CoV-2 S1 and RBD-specific IgG **(B)** and IgA levels **(C)** were measured by ELISA using pooled sera. Cumulative data from two experiments with n = 20 mice per group. Subsets of mice (n = 10–12) were sacrificed 2 weeks post-vaccination to evaluate mucosal immune responses. BAL samples were collected and analyzed individually for the quantification of SARS-CoV-2 S1 specific IgG and IgA levels **(D)** by ELISA. Neutralizing activity of immune serum [(at different time points) **(E)**] or BAL fluid **(F)** against SARS-CoV-2 was measured by MNT_50_. Positive correlation between neutralizing antibody response (Day 30) and S1-specific IgG response in immune serum of 5x10^10^ VP vaccinated animals **(G)**. Correlation analysis was performed with a two-tailed Pearson test. Secreted cytokine production by splenic T cells was measured by cytometric bead array kit **(H)**. Splenocytes were harvested from a subset of mice (n = 10–12) at 2 weeks post-vaccination and re-stimulated with S1 antigen for 72 hrs. Data points represent mean ± SEM of individual animal data. Error bars indicate mean with 95% CI (d, e & f). Statistical analysis was performed with nonparametric t-test: *P < 0.05; **P < 0.01.

Immunogenicity of BBV154 candidate vaccine was evaluated in inbred (BALB/c) and outbred (Swiss albino) mice (**Figure 2A**). Humoral and cellular immune responses were assessed in mice two-weeks post vaccination with either one-tenth, one quarter or half (1x10^10^, 2.5x10^10^ or 5x10^10^ VP respectively) of a human single dose (HSD). Single dose vaccination of BBV154 elicited systemic and mucosal immune response against SARS-CoV-2 spike as early as 14 days post vaccination (**Figure S4A**). On day 21 post-vaccination, significant spike-specific systemic IgG and IgA responses were detected in most of the vaccinated animals (p=<0.05) (**Figures 2B, C**), with comparable spike-specific IgG immune responses in BALB/c and Swiss Albino sera (Figure S4B). Mice vaccinated with 5 × 10^10^ VP had higher spike-specific antibody titers than those administered 1×10^10^ VP, demonstrating a dose-dependent response (**Figures 2B, C**). Additionally, substantial increases in serum IgG and IgA levels were observed on day 56 compared with day 21. Spike-specific antibodies were persistent and still measurable on day 70, ten weeks after vaccination, demonstrating durable immunity. IN immunization is known to stimulate mucosal IgA antibodies, providing a first line of defence against respiratory pathogens, and IN vaccination with BBV154 induced S1-specific IgG and IgA responses in the bronchoalveolar lavage (BAL) fluids (**Figure 2D**). As with the systemic IgG and IgA, dose-dependent pulmonary antibody responses were observed.

Vaccine-induced neutralizing antibody responses against SARS-CoV-2 NIV-2020-770 (containing the D614G mutation) (22) were assessed using a live virus microneutralization test (MNT_50_). Consistent with the spike-specific antibody responses, immune sera from BBV154 vaccinated mice neutralized SARS-CoV-2. Responses were dose-dependent: mice vaccinated with 5×10^10^ VP had higher neutralizing antibody titers (GMT = 264), than those administered 2.5×10^10^ VP (GMT = 76) which in turn were significantly higher (p=0.02) versus those administered 1×10^10^ VP (GMT = 45) (**Figure 2E**). Notably, these neutralizing antibody GMTs remained unchanged even after 56 days post vaccination. Further, the assessment of SARS-CoV-2 neutralizing antibodies in bronchoalveolar lavage (BAL) fluid of BBV154 immunized mice also displayed SARS-CoV-2 neutralizing antibodies in dose-dependent manner (**Figure 2F**). As expected, neither serum nor BAL fluid from placebo-treated mice exhibited any SARS-CoV-2 neutralizing activity. Further, the level of the neutralizing antibody response was well-correlated with S1-specific systemic IgG levels quantified in individual animals (Pearson r^2^ = 0.7504, **Figure 2G**), signifying that robust antibody responses to spike protein were allied with generation of potentially protective neutralizing antibodies.

Having observed a robust antibody response in vaccinated mice, we next examined the cell mediated immune (CMI) response activated *via* IN immunization. *Ex vivo* re-stimulation of splenocytes of vaccinated animals with S1 protein resulted in a significant induction of Th1 associated IFN-γ or TNF-α cytokines (p=0.007) (**Figure 2H**). Moreover, T-cells from the BBV154 immune animals produced modest levels of IL-10 and IL-4 when compared with T-cells from the placebo-treated mice. This indicates that IN vaccination of BBV154 induced Th1 mediated antiviral T-cell responses.

### Repeated-dose IN immunization of BBV154 elicits anti-spike humoral response

**Figure 3:**
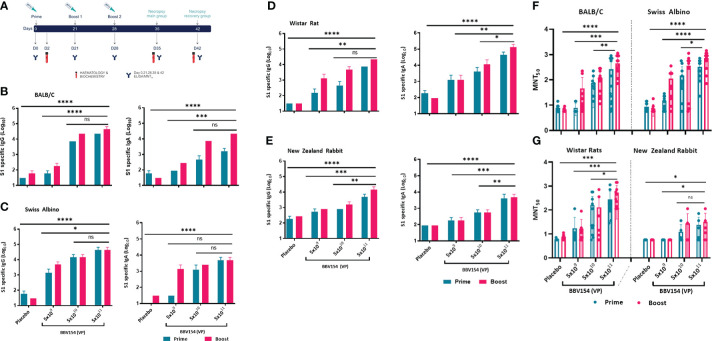
Candidate vaccine BBV154 elicits robust humoral immune responses in BALB/c, Swiss albino mice, Wistar rats and New Zealand White Rabbits. Schematic diagram of repeated dose administration of BBV154 to 6–8-week-old, male and female BALB/c or Swiss albino or Wistar rats (n = 10–12) or 10–12-week-old male and female New Zealand White rabbits (n = 6–8). Animals were immunized with placebo or three different concentrations of BBV154 vaccine candidate *via* the intranasal route in 50 μl (mice),100 μl (rat), and 200 μl (rabbit) on day 0 and boosted on day 21 (BALB/c & Rabbits) or 22 (Swiss albino and rats), and day 28 or 29 **(A)**. Antibody responses in sera of immunized animals at day 21 after priming or at day 28 or 29 after one-week post-boosting were evaluated **(B-E)**. SARS-CoV-2 S1-specific IgG (left panel) and IgA (right panel) levels were measured by ELISA using pooled sera of BALB/c **(B)**, Swiss albino **(C)**, Wistar rats **(D)** and New Zealand White Rabbits **(E)**. Neutralizing activity of immune serum against SARS-CoV-2 performed by MNT_50_ at day 21 after priming or at day 28 or 29 after one-week post-boosting **(F-G)**. Data points represent mean ± SEM of individual animal data. Statistical analysis was performed with nonparametric t-test: *P < 0.05; **P < 0.01; ***P < 0.001; ****P < 0.0001.

The immunogenicity and tolerability of clinical batch samples of BBV154 vaccine were evaluated in mice, Wistar rats and New Zealand White rabbits with a full human dose (N+1) regimen. Repeated doses of different concentrations of candidate vaccine (5x10^9^ VP [low-dose], 5x10^10^ VP [medium-dose], or 5x10^11^ VP [high-dose] per animal) were administered IN on days 0, 21 and 28 (**Figure 3A**). Serum samples were collected 21 days post-primary or pre-prime booster immunization and spike-specific IgG and IgA responses were evaluated by ELISA. Mice, rats and rabbits immunized with high- and medium-doses of vaccine elicited significantly higher IgG and IgA responses against purified S1 antigen than in the low-dose group (**Figures 3B–E**). Consistent with the spike binding antibody response, BBV154 vaccination elicited substantial increases in the SARS-CoV-2 specific neutralizing antibodies in mice, rats and rabbits (**Figures 3F, G**). Notably, boosting enhanced serum neutralization activity two- to four-fold in high-dose group (5x10^11^ VP) animals, with insignificant increases in rabbit immune sera (**Table 2**), whereas no neutralizing antibodies were detected in sera from placebo-treated animals after primary immunization or boosting.

**Table 2:** SARS-CoV-2 neutralizing antibody responses in serum following single or double intranasal administration of candidate vaccine BBV154. .

Animal Model	Dose (VP/animal)	Neutralizing Antibody Titers (MNT_50_)(mean)	
		Prime	Booster
**BALB/c Mice**	Placebo	7.6 ± 2.6	6.7 ± 1.3
	5x10^9^	7.8 ± 4.0	45.8 ± 78.3
	5x10^10^	76.6 ± 68.8	120.9 ± 97.8
	5x10^11^	264.8 ± 347.3	454.1 ± 356.9
**Swiss Albino Mice**	Placebo	8.6 ± 3.2	7.2 ± 3.5
	5x10^9^	13.9 ± 7.7	111.9 ± 111.6
	5x10^10^	146.6 ± 181.7	359.5 ± 366.8
	5x10^11^	325.1 ± 217.0	741.1 ± 434.5
**Wistar Rats**	Placebo	5.7 ± 0.4	7.4 ± 1.7
	5x10^9^	17.3 ± 31.8	16.7 ± 23.4
	5x10^10^	158.9 ± 208.5	128.2 ± 231.4
	5x10^11^	278.3 ± 338.0	545.2 ± 503.8
New Zealand Rabbit*	Placebo	5.7 ± 0.0	5.7 ± 0.0
	5x10^9^	5.7 ± 0.0	5.7 ± 0.0
	5x10^10^	12.4 ± 11.9	9.5 ± 9.4
	5x10^11^	23.9 ± 26.0	26.5 ± 41.4

Each group consisting of 10–12 animals; *Consisting of 4–6 animals; Limit of detection-5.6.

**Figure 4:**
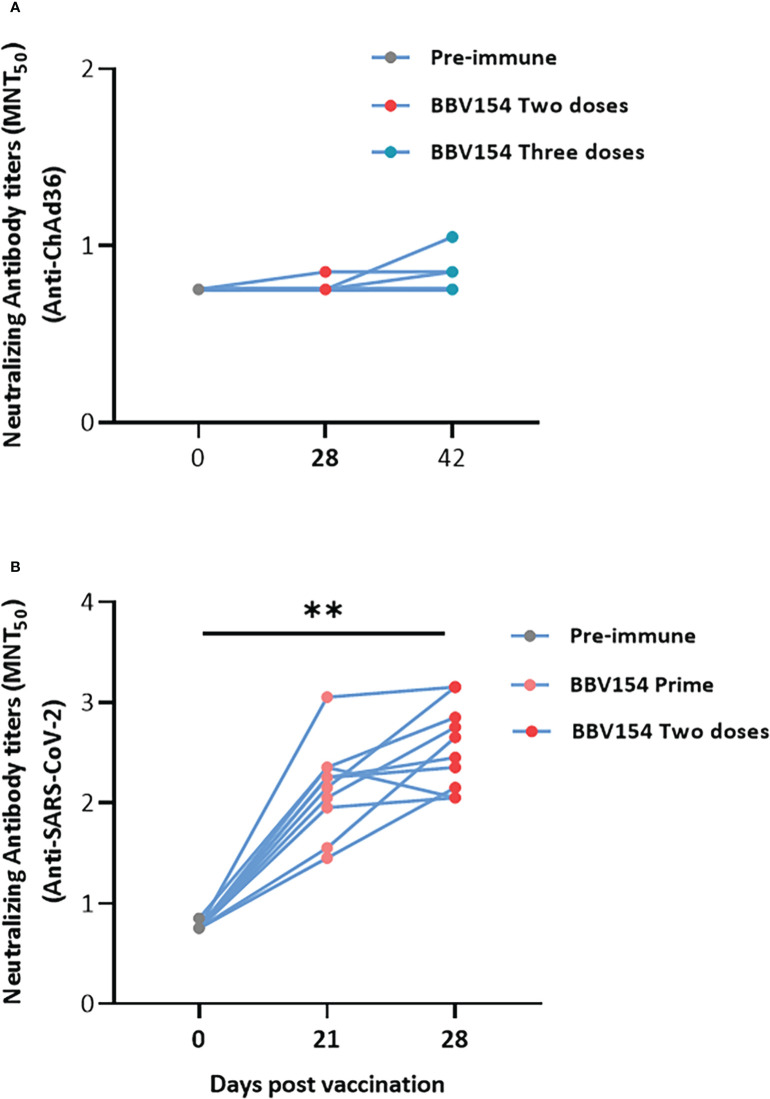
IN administration of BBV154 did not induce ChAd36 neutralizing antibodies even after repeated doses. Wistar rats (n = 10–12) were immunized IN with BBV154 vaccine as described in **Figure 3A**. Kinetics of anti-ChAd36 neutralizing antibodies in pre-and post-vaccinated immune sera of rats following three doses of 5×10^11^ VP of BBV154 **(A)**. Kinetics of anti-SARS-CoV-2 neutralizing antibodies in pre- and post-vaccination sera of rats following two doses of 5×10^11^ VP of BBV154 **(B)**. Serum samples were assessed using a MNT_50_ assay. Comparison between different time points were conducted using nonparametric t-test. **P < 0.01.

We then assessed the levels of anti-vector (ChAd36)-specific neutralizing antibodies in vaccinated animals. In line with a previous study (23), most of the animals did not produce the ChAd36 neutralizing antibodies, only 3 out of 12 vaccinated rat sera appearing to have low titers of anti-ChAd36 antibodies (**Figure 4A**). However, the immune sera derived from the same animals displayed significantly higher levels of SARS-CoV-2 virus neutralization activity compared with pre-immune sera (**Figure 4B**). Absent or insignificant titers of vector (ChAd36)-specific neutralizing antibodies following three doses of BBV154 implies that IN administration may offer an advantage for repeat vaccination using adenovirus-vectored vaccines.

### BBV154 immunogenicity in the young and aged hamsters

**Figure 5:**
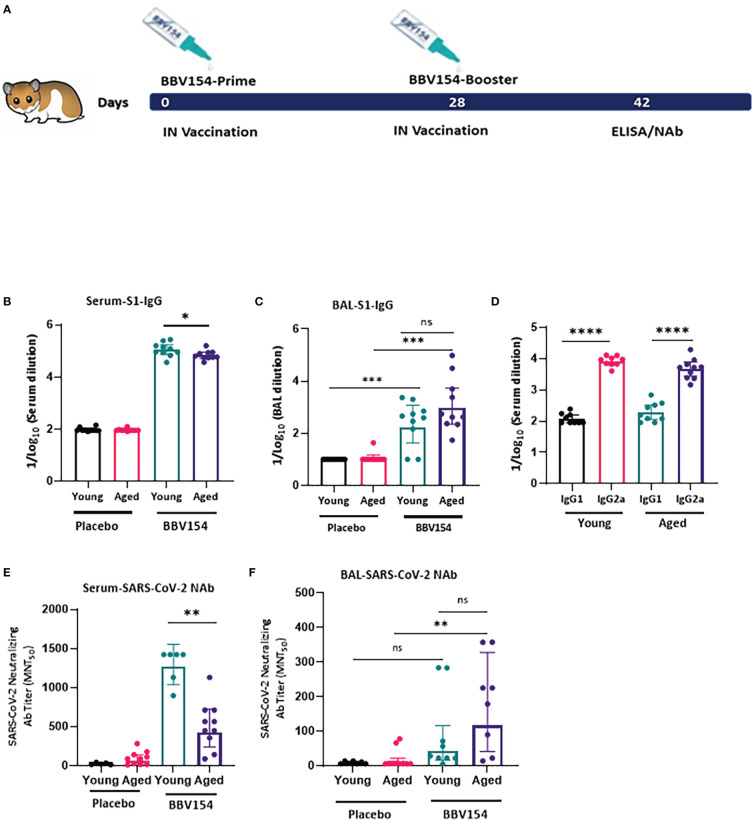
BBV154 induces strong systemic humoral immune responses in young versus aged Syrian hamsters. Young (9–11-weeks-old), and old (28–36-weeks-old) male and female Syrian hamsters (n=10) were immunized intranasally with placebo or 1x10^11^ VP of candidate vaccine BBV154 in 100 μl on day 0 and boosted on day 28 **(A)**. Antibody responses in sera of immunized animals were evaluated two weeks post-boosting. SARS-CoV-2 S1-specific IgG, in serum **(B)** and BAL **(C)**, serum IgG2a/IgG1 levels **(D)** were measured by ELISA using individual sera. Neutralizing activity of immune serum **(E)** and BAL **(F)** against SARS-CoV-2 was performed by MNT_50_. Significance was measured using nonparametric t-test: *P < 0.05; **P < 0.01; ***P < 0.001; ****P < 0.0001.

Syrian hamster (Mesocricetus auratus) has been used in diverse research studies on COVID-19 vaccine studies. Hamsters are naturally susceptible to SARS-CoV-2 infection, and intranasal inoculation mimics the human disease in comparison to other animals (24). Aged and male hamsters develop more severe disease, mimicking COVID-19 in humans (25). Aging is accompanied by a diminishing adaptive immune response (26). We explored age-dependent differences in the immunogenicity of BBV154 candidate vaccine in young (9–11 weeks) or aged (28–36 weeks) Syrian Hamsters (**Figure 5A**). Consistent with earlier preclinical studies by Bricker et al. (17), following vaccination significant spike-specific IgM [systemic (**Figure S5A**)] and IgG (systemic and mucosal) responses were detected in most of the vaccinated hamsters. Further, BBV154-immunized young animals showed a moderate but significant increase in levels of spike-specific IgG compared with older animals (**Figure 5B**), whereas both young and old animals presented comparable mucosal IgG responses (**Figure 5C**). BBV154 vaccination in young and old animals elicited a lower IgG1 antibody response than IgG2, indicating Th1 phenotype, with IgG2a/IgG1 ratios greater than 1 (**Figure 5D**). Due to non-availability of commercial Anti-hamster-IgA secondary antibodies, IgA responses were not analysed. As with spike-specific binding antibodies, BBV154 immune sera from young animals had much higher levels of SARS-CoV-2 neutralizing activity than immune sera from old animals (**Figure 5E**). Levels of neutralizing antibodies in BAL were found to be comparable in young and old animals (**Figure 5F**).

### Systemic prime-intranasal boost strategy augments BBV154 vaccine efficacy

**Figure 6:**
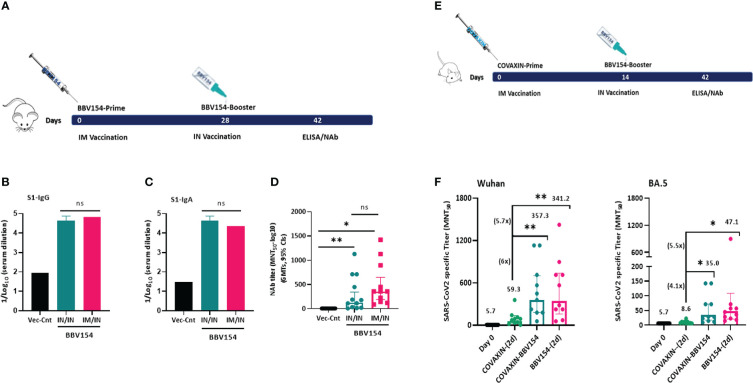
Comparison of humoral immune responses induced by a systemic prime-intranasal boost strategy by homologous or heterologous COVID-19 vaccines. 5–6-week-old BALB/c male and female mice (n=10-12) were primed intranasally or intramuscularly with 5x10^10^ VP of Vector control or BBV154 on day 0 and boosted IN on day 28 **(A)**. Two-weeks post-booster vaccination SARS-CoV-2 spike specific IgG **(B)** and IgA **(C)** were assessed by ELISA, SARS-CoV-2 neutralizing antibodies by MNT_50_ **(D)**. 6–8-week-old male and female Wistar rats (n = 10) in three groups were immunized with homologous BBV154 prime and BBV154 booster (one-fifth of a human single dose) or COVAXIN prime and COVAXIN [one-fifth of a human single dose) booster or heterologous (COVAXIN prime and BBV154 booster) regimens on day 0 and 14 **(E)** four-weeks post-booster vaccination neutralizing activity of immune serum **(F)** against SARS-CoV-2 ancestral (Wuhan) or omicron variant (BA.5) was performed by MNT_50._ The numbers in parentheses indicate the GMT and fold change in neutralization titer against COVAXIN homologous group compared with the heterologous (COVAXIN/BBV154) or BBV154 homologous groups. Error bars indicate mean with 95% CI. Statistical analysis was performed with nonparametric t-test: *P < 0.05; **P < 0.01.

Effective vaccination strategies need not be restricted to one route of administration alone; several vaccine studies have demonstrated that memory cells primed by parenteral vaccination can be “pulled” into mucosal sites by successive mucosal vaccination (27–29). To test this, a group of naive BALB/c mice were primed by IM vaccination with BBV154 on day 0 followed by an IN booster on day 28, and the immune responses were compared with mice which received two IN BBV154 vaccinations (**Figure 6A**). The IM prime-IN booster vaccination induced similar levels of S1-specific IgG and IgA and slightly increased but not statistically significant neutralizing antibody titers (p=0.25) compared with the IN-prime and IN-boost mice (**Figures 6B–D**). To test this further, a heterologous vaccination study was conducted with COVAXIN^®^ IM priming followed by IN BBV154 booster and the resulting immune responses were compared with homologous COVAXIN/COVAXIN or BBV154/BBV154 immune regimens. BBV154 IN booster in COVAXIN-primed rabbits elicited S1-binding and SARS-CoV-2 neutralizing antibody response which was four-eight times stronger than that in homologous COVAXIN/COVAXIN immune model (**Figures S6B–D**). Emergence of SARS-CoV-2 variants of concern (VOC) has raised concerns about the breadth and durability of neutralising antibody responses (30). To address this, homologous and heterologous immune sera from Wister rats was subjected to SARS-CoV-2 ancestral (Wuhan) and omicron variant neutralization studies (**Figure 6E**). Heterologous vaccination elicited significantly higher neutralizing antibody titers of 6 and 4.1-fold against SARS CoV-2 ancestral (Wuhan) and omicron variant (BA.5) respectively (**Figure 6F**) as compared with the homologous COVAXIN/COVAXIN immune model. Notably, BBV154 two-dose immune sera also induced higher neutralizing antibody titers of 5.7 and 5.5-fold against SARS CoV-2 ancestral (Wuhan) and omicron variant (BA.5) as compared to homologous COVAXIN/COVAXIN immune model (**Figure 6F**).

## Discussion

There has been a great interest in the intranasal vaccine approach to contain COVID-19 (5, 29). Earlier studies have demonstrated that the IN delivery of influenza vaccines generates sterilizing immunity and prevents the transmission of influenza A virus (31, 32). Similarly, IN delivery of replication-defective adenovirus-vectored vaccines simulate the route of natural infection which leads to stimulation of efficient humoral and cellular immunity, both systemically and mucosally, whereas vaccines administered parenterally primarily stimulate only systemic immune responses (23, 33). Correspondingly, IN administration of a ChAd-vectored SARS-CoV-2 vaccine elicits highly potent immune responses in relevant animal models including mice, hamsters and rhesus macaques (16–18, 34).

In this study, we assessed the safety and immunogenicity of an intranasal candidate vaccine, BBV154, a ChAd-vectored vaccine expressing a pre-fusion stabilized spike protein of SARS-CoV-2. Several features distinguish BBV154 from ChAdOx1 nCoV-19, a chimpanzee Ad-23-based SARS-CoV-2 vaccine that has received emergency use authorization approval in several countries for IM administration in humans. BBV154 is derived from a simian Ad36 serotype with further deletions in the backbone to enhance production of virus (GenBank: FJ025917.1) (35). Multiple tests were performed to characterize the BBV154 vaccine candidate; the presence of a spike expression cassette in the BBV154 genome was confirmed by conventional PCR and next generation sequencing (data not shown). The expression of spike protein in BBV154-infected cells was detected by western blotting and immunocytochemistry approaches. The downstream purified BBV154 protein profile was comparable to adenovirus reference material obtained from ATCC. Quantification of RCA in the adenovirus vector-based vaccine preparation is an important safety parameter; the US FDA requirements are <1 RCA in 3 x 10^10^ VP for clinical purposes (36). In the BBV154 vaccine preparation we could not detect RCA even after passaging it three times in A549 cells, indicating that BBV154 is safe to use in clinical studies.

**Figure 7:**
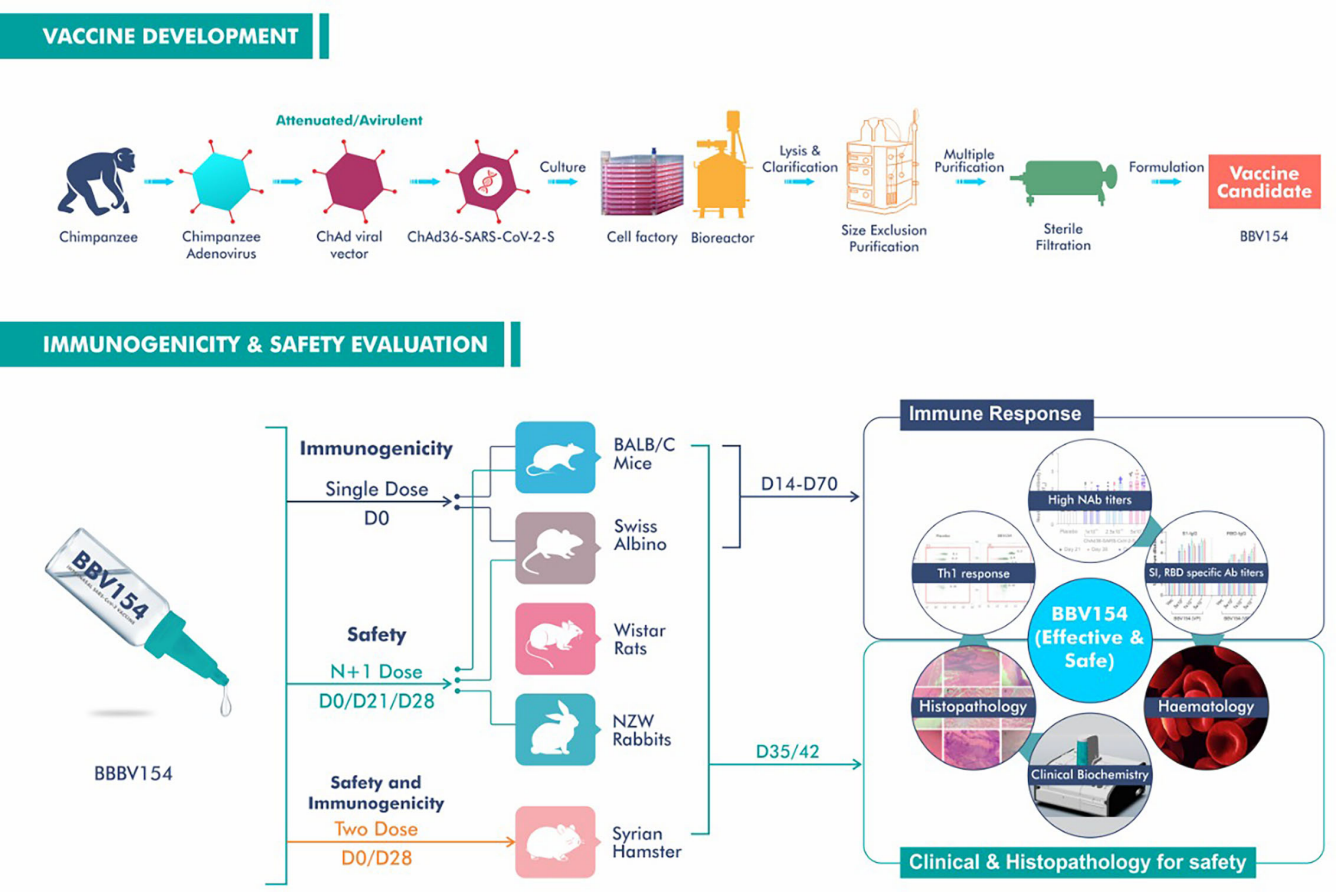
Schematic representation of manufacturing process, immunogenicity and safety evaluation studies of BBV154 vaccine candidate.

As a further step towards the clinical development, we established the safety and immunogenicity of the BBV154 vaccine candidate *via* IN administration in rodent and non-rodent animal models (**Figure 7**). Our safety evaluations in rodents and non-rodents have demonstrated that there were no local or systemic adverse effects attributable to the vaccine following repeated intranasal administrations. Findings from *in vivo* observations, clinical pathology, necropsy data and histopathological evaluations have shown that the vaccine has a favourable safety profile in all the preclinical species tested. Platelet count, prothrombin time and activated partial thromboplastin time (APTT) in vaccinated animals were well within the range and comparable with the concurrent control group. These observations showing that BBV154 nasal administration did not lead to thrombosis or thrombocytopenia are important as these conditions have been observed in healthcare workers and others who received IM injections of ChAdOx1 nCoV-19 vaccine (37, 38).

The immune responses we observed are comparable with earlier results (16, 17, 39). Single IN immunization of BBV154 with one tenth, one quarter or half the human dose elicited systemic and mucosal spike-specific binding antibody and SARS-CoV-2 neutralizing antibodies coupled with Th1 immune response. Nasal delivery of BBV154 vaccine formulation also elicited significant levels of spike-specific mucosal (secretory) IgA which is essential and involved in early virus neutralization of SARS-CoV-2 as observed in convalescent individuals (40, 41). Spike-specific serum IgG and IgA levels continued to be detected up to day 70, ten weeks after vaccination, demonstrating BBV154 has the potential to provide long-lasting humoral immunity. BBV154 induced significant amounts of IFNγ, a key cytokine for several antiviral responses including those against SARS-CoV-2 (42). Immune sera and BAL from vaccinated animals presented enhanced neutralizing antibody titers, which has been established as a correlate of protection in vaccinated non-human primates as well as SARS-CoV-2 recovered individuals (43). Aging is accompanied by changes in the immune system, adaptive immune responses are particularly diminished in older individuals making them less responsive to the majority of existing vaccines (26). BBV154 vaccination elicited robust immune responses in the young hamsters but not in the aged animals indicating that responses to BBV154 vaccine are likely age-dependent, at least in hamsters.

Immunity against virus capsid proteins severely reduces the effectiveness of adenovirus-vectored vaccines, particularly with parenteral administration, and to compensate the pre-existing immunity relatively high dosage of Ad5 vectored Ebola virus vaccine was used in seropositive individuals (7, 44). In contrast, pre-clinical studies of mucosal immunization of recombinant (human and chimpanzee) adenovirus-based vaccines circumvented the pre-existing immunity and conferred sufficient protection against challenge with variety of pathogens (23, 45). Consequently, the absence or insignificant levels of ChAd36 neutralizing antibodies following repeated doses of BBV154 vaccination implies that IN administration may offer an advantage even after repeated vaccination of adenovirus-vectored vaccines. To meet the challenges from VOC with high transmissibility and enhanced tropism in upper respiratory tract and ability to evade the vaccine-induced immunity (46–48), many countries have initiated booster doses to a vaccinated population that has completed a primary vaccination series (49, 50). Immunogenicity data of heterologous schedules are available for a wider range of COVID-19 vaccine combinations (51). Vaccine efficacy studies have largely reported on heterologous primary schedules involving ChAdOx1 nCoV-19 followed by an mRNA vaccine, showed marginally greater vaccine efficacy than that of homologous modules (52, 53). However, the current COVID-19 vaccines, including mRNA, are not designed to elicit the mucosal immunity that is critical for blocking the SARS-CoV-2 infection and transmission (4, 54), leading to a risk of transmission of virus from vaccinated individuals and continuous emergence of VOC and breakthrough infections (55). Thus, effective vaccination strategies should not be restricted to a single route of administration, and in addition to IM only vaccines, inclusion of systemic prime and intranasal boost strategies may enhance the vaccine efficacy (29, 56). Accordingly, COVAXIN priming followed by BBV154 IN-booster elicited superior immune responses, with an acceptable reactogenicity profile comparable to the homologous COVAXIN/COVAXIN or BBV154/BBV154 vaccination model. Jinyi Tang et al, demonstrated that mucosal booster vaccination is needed to establish robust sterilizing immunity in the respiratory tract against SARS-CoV-2, including infection by omicron sublineage and future VOCs (57). Similarly, BBV154 intranasal booster delivery in COVAXIN primed animals induced significantly higher neutralizing antibodies (p=0.005 & 0.01) against SARS-CoV-2 ancestor as well as omicron variant as compared to homologous COVAXIN/COVAXIN. The broad epitope protection from an inactivated vaccine (COVAXIN) (58) and the heightened cell mediated responses/mucosal protection conferred from an adeno-vectored IN vaccine (BBV154) may augment the efficacy of heterologous prime-boost schedules. Our findings support the necessity of inclusion of intranasal vaccine BBV154 to establish robust sterilizing immunity in the respiratory tract as a standalone or booster dose to curtail the emergence of SARS-CoV-2 variants and pandemic spread.

A major limitation of this study is the lack of protective efficacy results conferred from GMP lots of BBV 154, though, our collaborator has published a series of protection efficacy studies in mice, hamster and macaques. Additional live challenge studies must be conducted to demonstrate the transmission blockage following BBV154 IN vaccination.

In summary, our studies demonstrated that, IN administration of BBV154 presented excellent safety profiles in terms of pathology and biochemical analysis and elicited robust mucosal and systemic humoral immune responses coupled with Th1-mediated immune responses. Heterologous prime-boost vaccination with intramuscular (IM) COVAXIN-prime followed by BBV154 intranasal elicited superior and cross variant protective immune responses. BBV154 has now completed both homologous and heterologous combination schedules of human phase 3 clinical trials and received the restricted emergency use approval (in those aged above 18 years) from the Drugs Controller General of India (DCGI).

## Methods

### Viruses and cells

The chimpanzee adenovirus 36 vector encoding SARS-CoV-2 pre-fusion stabilized spike protein (ChAd36-SARS-CoV-2-S) and the chimpanzee adenovirus 36 vector control (ChAd36-Control) were obtained from Curiel and Diamond’s Labs, Washington University in St Louis, USA (16). The adenovirus reference material; wild-type human Adenovirus type 5 (Ad5) (Catalogue No: VR1516) was procured from ATCC, USA. The HEK cells were obtained from Microbix, Canada and were propagated in DMEM (Gibco, USA), supplemented with 5% heat-inactivated fetal bovine serum (FBS; Gibco), and neomycin (Gibco, USA). The HEK cells were maintained at 37°C with 5% CO_2_. These cells were used as a substrate for the production of the BBV154 and in QC testing.

The human adenocarcinoma cells (A549, ATCC^®^ CCL-185™, USA) was obtained from ATCC and propagated in Eagle’s minimum essential medium (Gibco, USA), supplemented with 10% heat-inactivated fetal bovine serum (FBS; Gibco), and 1 x penicillin-streptomycin (Gibco, USA). These cells were used for the estimation of replication competent adenovirus (RCA) in the BBV154 samples.

For large-scale production of candidate vaccine BBV154, the E1 complimenting (HEK 293) cells were infected optimal MOI and the cells harvested between 60to 72 hours post infection and cells were lysed, DNA fragmented and clarified and sterile filtered with depth filters. These samples were further subjected to size exclusion chromatography which is followed by approximately 3 to 5 times concentration to produce the drug substance.

### Plasmids

The genomic plasmids of ChAd36-SARS-CoV-2-S and ChAd36-control were obtained from Curiel and Diamond’s Labs, Washington University in St Louis, USA and propagated in *E. coli* DH10B. These were used as a positive control for the estimation of genome copies by quantitative polymerase chain assay (qPCR).

### Antibodies

The rabbit polyclonal RBD antibody was raised against bacterial expressed receptor binding domain (331 – 520 aa) of SARS-CoV-2 spike and was used for western blotting. Purified S1 protein (expressed in 293 cells) was obtained from Syngene International Limited (Bangalore, India) and rabbits were immunized to obtain rabbit polyclonal S1 antibody. This antibody was used for immunocytochemistry assays. Neutralizing antibodies against ChAd-control vector were raised against an ultracentrifuge pellet of ChAd-control and were used as a positive control in the ChAd neutralization assay.

### Transgene expression cassette in the ChAd-S genome

The PCR primers bound to the ChAd genome flanking the expression cassette were used to demonstrate the presence of the expression cassette of expected product size. The details of the primers used in this study are given in **Table S1**.

### Virus particle estimation

The purified BBV154 virus particles were disassembled with 0.1% sodium dodecyl sulfate and enumerated as described previously (59). The optical density (OD) was measured at 260 nm and the virus particles were extrapolated from the formula: Virus particles/mL =OD260 x dilution factor x 1.1 x 10^12^.

### Quantitative polymerase chain assay (qPCR)

A qPCR assay was standardized for the estimation of the adenovirus genome copies in BBV154 samples. Both hexon and the spike genes were targeted with primer probes with 5’ FAM fluorophore and 3’ BHQ quencher. The genomic plasmids of ChAd36-SARS-CoV-2-S was used as the standard to derive the log copies/mL of the BBV154 sample. The details of the primers and probes that were used for the qPCR assay are listed in **Table S1**.

### Tissue culture infective dose 50 (TCID_50_)

Infectious titers of the ChAd samples were determined by TCID_50_. Ten-fold serial dilutions of samples from 10^-1^ to 10^-10^ were co-seeded along with 10^4^ HEK cells in 96-well plates and incubated for 10–12 days at 37°C with 5% CO_2_. Six replicates of each dilution were observed under microscope and the wells showing presence of cytopathic effect (CPE) were considered positive and the wells with no visible CPE were considered as negative. The virus titer was calculated by the Reed and Muench method (60).

### Immunocytochemistry

Approximately 10^5^ number of HEK cells were seeded per well in a 12-well plate and incubated for 16 h at 37°C with 5% CO_2_. Post-incubation the HEK cells were incubated with 100 µL of 10^-1^ and 10^-2^ dilutions of BBV154 vector for 45 min at 37°C with 5% CO_2_. Inoculum was removed and 1 mL of MEM with 1% FBS was added to each well and incubated at 37°C with 5% CO_2_ for 48 h. The spent medium was removed from the cells and ice-cold methanol was added to the cells and incubated at -20°C for 20 min. The fixed cells were washed with PBS and incubated with rabbit polyclonal S1 antibody. Anti-rabbit IgG peroxidase conjugate was used as the secondary antibody (Sigma-Aldrich, USA). Insoluble chromogen; 3-amino-9-ethylcarbazole (AEC) was used for the detection of spike in the BBV154 infected HEK cells. Cells expressing spike are expected to be stained with AEC as dark pink cells.

### Western blotting

BBV154 vector-mediated expression of SARS-CoV2 spike in HEK cells was analysed by western blotting. Briefly, the HEK cells were either infected with BBV154 at a multiplicity of infection (MOI) of 5 or were left uninfected. The cells were harvested 24 h post infection, and extracts were prepared in lysis buffer (50mM Tris-HCl [pH 7.5], 100 mM NaCl, 1% (3-((3-cholamidopropyl) dimethylammonio)-1-propanesulfonate (CHAPS), and 1mM phenylmethylsulfonyl fluoride). The lysates were subjected to 10% sodium dodecyl sulfate–polyacrylamide gel electrophoresis (SDS-PAGE) and transferred onto polyvinylidene fluoride (PVDF) membrane for 90 min at 250 mA using semi-dry transblot apparatus (Thermo Fisher Scientific, USA). Membranes were blocked with 5% skimmed milk powder in PBS (SMP) overnight at 4°C, incubated with rabbit polyclonal and rabbit polyclonal antibody or RBD specific antibody at a dilution of 1:1,000 in 3% SMP at room temperature for 1 h and washed three times with PBS containing 0.05% Tween-20 (PBST). The membranes were incubated with anti-rabbit IgG whole molecule-peroxidase conjugate (Sigma-Aldrich, USA) at 1:5,000 dilution in 3% SMP at 37°C for 1 h and washed three times with PBST, and once with PBS. Luminol-based enhanced chemiluminescence reagent (SignalFire™ ECL reagent, Cell Signaling Technology, USA) was used to develop the luminescence signal which was captured on X-ray film. The exposed X-ray films were developed and fixed.

### Growth kinetics of BBV154

The HEK cells were infected with 0.25, 0.5, 1, 2 and 3 MOI of BBV154 vector. Samples were collected at 12 hours interval from 12 to 82 hours post infection. These samples at each time point were subjected to qPCR or 50% tissue culture infective dose (TCID_50_) to estimate the genome copies/mL or TCID_50_/mL.

### Test for detection of Replication Competent Adenovirus (RCA)

The samples were passaged three times in A549 cells followed by 50% tissue culture infective dose (TCID_50_) in A549 cells. Briefly, A549 cells were seeded in T 25cm^2^ flasks and infected with 1 mL of BBV154 samples alone or or 10 TCID of Ad5 alone or BBV154 samples spiked with 1 TCID of Ad5 or BBV154 samples spiked with 10 TCID of Ad5. Forty-eight hours post-incubation at 37°C with 5% CO_2_, the flasks were frozen at -80°C and were subjected to three freeze-thaw cycles. The contents of the flasks were collected and centrifuged at 16000 g for 10 min at 4°C. The supernatants were passaged two more times in A549 cells and the RCA in the samples were estimated by TCID_50_ in A549 cells or HEK cells (60).

### Manufacture of BBV154 vaccine candidate

The HEK cell master and working cell banks were prepared in a GMP facility. Working cell bank was used for the preparation of the BBV154 master and working virus banks in a GMP facility. The cell banks and the virus banks were stored at the banking facility and characterized as per ICH guidelines for identity, safety and purity. The GMP production of virus bulk drug substance was initiated by growing HEK working cell bank in T-175 cm^2^ flasks and expanding them in CellSTACK^®^ 1, 10 and 40 stacks (Corning Inc., USA). The HEK cells were propagated up to a cell density of 1400 million cells/CS40 and were infected with working virus bank of BBV154 at 1 MOI. Infected cells along with the spent media were harvested when >80% cytopathic effect was reached. The adenovirus particles were released by treatment with lysis buffer for one hour at room temperature. The lysate was clarified by depth filtration to remove cell debris. The clarified lysate was then concentrated using ultrafiltration membranes and buffer exchanged with phosphate buffered saline (pH 7.4). Retentate sample was passed through a size-exclusion matrix to remove impurities. Flow-through from the size-exclusion chromatography was further concentrated, buffer exchanged and passed through a 0.2 micron filter to obtain the sterile drug substance. The BBV154 drug product was produced by diluting the drug substance to required concentration of total virus particle with formulation buffer and distributed in vials.

### Intranasal administration

Studies were performed following both national and international guidelines in compliance with OECD Principles of GLP (61). Four animal models (BALB/c and Swiss Albino mice, Wistar Rats, Syrian hamster and New Zealand White Rabbits) were randomly assigned into different treatment groups: the control group (placebo), low dose group (5x10^9^ VP) medium dose group (5x10^10^ VP) and high-dose group (5x10^11^ VP) were immunized *via* the intranasal route with three doses (N+1) of vaccine on days 0, 21 and 28. Nasal administrations were performed under anaesthesia induced and maintained with ketamine hydrochloride and xylazine. All animals were observed for treatment-related adverse effects and mortality during the experimental period. Animals were bled at different intervals for detailed clinical pathology investigations. Animals were euthanized either on day 30 (main groups) or on day 42 (recovery groups) and necropsied, and organs were evaluated for macroscopic and microscopic findings.

### Heterologous prim-boost vaccination

To evaluate the immunogenicity of the heterologous BBV154 prime-boost regimens, six-to eight-week-old male and female BALB/c mice (n=10 per group) in two groups were anesthetized and immunized IN-prime and IN-boost (IN/IN) or IM-prime and IN-boost (IM/IN) with BBV154 of 5x10^10^ VP/animal on days 0 and 28. Two-weeks post-booster vaccination SARS-CoV-2 spike specific serum IgG, IgA and NAb were assessed. To assess the immunogenicity of the heterologous COVAXIN/BBV154 prime-boost regimens, 10–12-week-old male and female New Zealand White Rabbits (n = 4) in two groups were immunized with heterologous [COVAXIN prime (6µg/animal) and BBV154 booster (1× 10^11^ VP) or homologous (COVAXIN prime and COVAXIN booster) regimens on days 0 and 14 or 28. Two-weeks post-booster vaccination SARS-CoV-2 spike specific serum IgG, IgA and NAb were assessed. In another experiment, 6–8-week-old male and female Wistar rats (n = 10) in three groups were immunized with homologous BBV154 prime and BBV154 booster (one-fifth of a human single dose) or COVAXIN prime and COVAXIN [one-fifth of a human single dose) booster or heterologous (COVAXIN prime and BBV154 booster) regimens on day 0 and 14 four-weeks post-booster vaccination neutralizing activity of immune serum against SARS-CoV-2 ancestral (Wuhan) or omicron variant (BA.5) was performed by MNT_50._

### Antibody quantification

SARS-CoV-2 spike specific IgM, IgG, IgA, IgG1 and IgG2/IgG3 were measured by ELISA according to standard protocols. Briefly, microtiter plates were coated with S1 (Syngene International, India), receptor binding domain (RBD) (Genetex, India) at a concentration of 2 μg/ml, 80 μL/well in PBS pH 7.4 overnight at 4°C. The plates were then washed three times with 200 µL/well of PBS containing 0.5% SMP and 0.002% tween-20 (Sigma-Aldrich, India). Plates were then blocked at 37°C for 1 hour with 200 µL/well PBS containing 3% SMP. Serially diluted pooled or individual sera or BAL samples were added to the plates (100 µL/well) and incubated for 1 hours at 37°C. Secondary antibodies anti-mouse IgG and IgA HRP (Sigma-Aldrich, India) for mouse sera/BAL samples, anti-rat IgG HRP (Thermo Fisher Scientific, India) and anti-rat IgA HRP (Abcam, UK) for rat and anti-rabbit IgG HRP (Sigma-Aldrich, India) and IgA HRP (Abcam, UK) for rabbit samples were used. Goat anti-Hamster IgG (H+L) Secondary Antibody, HRP (Invitrogen), Rabbit Anti-Syrian Hamster IgM Secondary Antibody (Life Span Biosciences), Mouse Anti-Hamster IgG1-HRP and Mouse Anti Hamster IG2/IgG3 HRP (Southern Biotech) antibodies were used against hamster serum or BAL samples. Tetra (3,3′,5,5′) methylbenzidine (Denovo BioLabs Pvt Ltd., India) substrate was added. All samples were read at 450 nm using a microplate reader (iMark, Biorad) following a 5 second mixing. The end-point serum dilution was calculated with three times the standard deviation of the mean optical density (OD) value for pre-immune sera.

### *Ex-Vivo* splenocyte activation and cytokine analysis

On day 14 after primary immunization, spleen cells were harvested and diluted in medium to a concentration of 1 × 10^6^ cells/mL. Splenic T cells (10^6^) were incubated with 1 μg/ml of S1 protein for 72 hours at 37°C with 5% CO_2._ The secretion of multiple Th1, Th2 and Th17 cytokine profiles of ex-vivo stimulated splenocytes of placebo or vaccinated mice were analysed using Cytometric Bead Array Mouse Th1/Th2/Th17 Cytokine Kit (BD Bioscience, USA). Briefly, 10 µL of each capture bead was incubated with 50 μL of cell supernatant or standards along with 50 μL of phycoerythrin (PE) detection reagent consecutively to each assay tube and incubated for 2 hours at room temperature. The samples were washed with 1 mL of wash buffer and centrifuged at 200 g for 5 min. The bead pellet was re-suspended in wash buffer. All samples were acquired using Attune NxT Flow cytometer (Thermofisher), data analyzed using Attune NxT 4.2 software and FCS express software.

### Virus neutralization assay using SARS-CoV-2 or ChAd-SARS-CoV-2-S

SARS-CoV-2 strains [Wuhan (D614G) and Omicron variant (BA.5)] were obtained from National Institute of Virology (NIV), Pune, India. Both strains were adapted to a highly characterized GMP Vero cell platform, amplified to produce the master and working virus bank. The master virus bank was well characterized based on WHO Technical Report Series guideline. Virus propagation and characterization was performed in the bio-safety level-3 (BSL-3) facility using bioreactors. Wild-type or omicron variant virus neutralising antibody titres in serum and BAL samples were analysed with a microneutralisation test (MNT50) in Biosafety level 3 (BSL-3) testing facility at Bharat Biotech in a masked manner. The serum and BAL fluids were inactivated in a water bath at 56°C for 30 min. The heat-inactivated samples were serially two-fold diluted from 1:8 to 1:4096, and incubated with an equal volume of solution containing 100 CCID_50_ of SARS-CoV-2 or 100 TCID_50_ of ChAd-SARS-CoV-2-S viruses. After neutralization in a 37°C incubator for 1 hour, a 1.0 x 10^5^/mL cell suspension (Vero cells for SARS-CoV-2 and HEK cells for ChAd-SARS-CoV-2-S) was added to the wells (0.1 mL/well) and cultured in a CO_2_ incubator at 37°C for 3–5 days for SARS-CoV-2 and 10–12 days for ChAd-SARS-CoV-2-S. The neutralization end-point was defined as the highest dilution of serum that can protect 50% of the cells from infection following a challenge with 100 CCID_50_ SARS-CoV-2 or 100 TCID_50_ of ChAd-SARS-CoV-2-S virus. The titers were determined by the Karber method (62). Neutralization antibody potency of < 1:20 is negative, while that of > 1:20 is positive.

## Data availability statement

The original contributions presented in the study are included in the article/**Supplementary Material**. Further inquiries can be directed to the corresponding author.

## Ethics statement

The animal study was reviewed and approved by Institutional Animal Ethics Committee (IAEC).

## Author contributions

All authors meet the International Committee for Medical Editors criteria for authorship and have no conflicts to disclose. RS led the immunogenicity and safety preclinical experiments. KV designed prime-boost heterologous studies and data analysis. RE, SP and KV led the manufacturing and quality control efforts. RS acquired, analysed and interpreted the data and wrote the manuscript. RE and KV reviewed the manuscript. All authors contributed to the article and approved the submitted version.
