# Supplementary material for: Preclinical evaluation of safety and immunogenicity of a primary series intranasal COVID-19 vaccine candidate (BBV154) and humoral immunogenicity evaluation of a heterologous prime-boost strategy with COVAXIN (BBV152)
Source: Front Immunol. 2022 Dec 8;13:1063679. doi: 10.3389/fimmu.2022.1063679 (PMC9773076; doi:10.3389/fimmu.2022.1063679)
Supplement: Supplementary file 1 [file DataSheet_1.docx]

**Supplementary Material**

**Preclinical evaluation of safety and immunogenicity of a primary series intranasal COVID-19 vaccine candidate (BBV154), and humoral immunogenicity evaluation of a heterologous prime-boost strategy with COVAXIN (BBV152)**

Raju Sunagar, Sai D Prasad, Raches Ella and Krishna Mohan Vadrevu*

**Affiliation**

Bharat Biotech International Ltd, Hyderabad, India

*** Corresponding author:**

Dr. Krishna Mohan Vadrevu

Bharat Biotech International Ltd, Hyderabad (BBIL), Telangana - 500 078, India;

[kmohan@bharatbiotech.com](mailto:kmohan@bharatbiotech.com)

# **Pre-Clinical Toxicity Studies**

During the development of new vaccine, the purpose of pre-clinical studies is to provide safety information to support the clinical development and licensure of the product. The Preclinical study designs had taken into account the intended clinical use, route of administration, dose levels and immunization schedule in humans. Pathology phase of repeated dose toxicity studies were performed at RCC Laboratories India Pvt Ltd, Hyderabad which is an OECD GLP certified and AAALAC accredited facility as per Schedule Y, ICH and WHO guidelines following GLP standards.

## **Test item:** BBV 154 vaccine candidate was used for the pre-clinical studies which represented the vaccine formulation that is intended for clinical use. The test item was administered at three doses viz., 5 x 10^9^ VP/animal, 5 x 10^10^ VP/animal and 5 x 10^11^ VP/animal as low-, mid- and high-dose, respectively. A placebo group administered with vaccine excipients served as control group. Recovery groups for placebo, mid and high doses were also maintained in the studies.

## **Test system:** The test system *viz*., Wistar rats, Swiss albino mice, BALB/c mice (SPF animals) and New Zealand White rabbits were sourced from Committee for the Purpose of Control and Supervision of Experiments on Animals (CPCSEA) approved vendor and these test systems were selected as per the recommendations of WHO guidelines (2013) and Schedule Y (2019). Young adult animals were used and the average body weight ranged from 157 - 250 g for Wistar rats, 11 - 17 g for Swiss albino mice, 14 - 17 g for BALB/c mice and ~ 2 kg in both males and females for New Zealand White (NZW) rabbits. The studies were conducted in equal number of male and female animals.

## **Animal Husbandry Practices:** The animal rooms were air-conditioned with adequate air changes (12 - 15) per hour and provided with a light cycle of 12 hours light and 12 hours dark. The room temperature was maintained in the range of 22 ± 3°C (rat and mice), 20 ± 3°C (rabbits) and relative humidity between 30 and 70% and it was continuously monitored. Animals were housed in cages with dimensions adhering to CPCSEA guidance. Maintenance Diet and UV Purified water were provided *ad libitum*.

## **Ethical Approvals:** The studies were conducted post approval from Institutional Animal Ethics Committee (IAEC) and Institutional Biosafety Committee (IBSC) following all ethical practices as laid down in the guidelines for animal care and biosafety.

## **Treatment regimen:** The dose volume of 100 µL/animal (split dose; 50 µL/nostril) was administered by intranasal route in the four *in vivo* models on day 0, 21 and 28 (N+1) with full Human Single Dose (HSD)**.** The main group animals from placebo, mid- and high-dose groups were sacrificed 2 days, low-dose animals were sacrificed 7 days and recovery groups were sacrificed 14 days post last dose.

**In-life Observations:** All animals were observed twice daily for mortality. Clinical signs were recorded twice a day from day 0 to 2 and once daily thereafter. The cage side observations included changes in skin, fur, eyes and mucous membranes and clinical signs observed for oedema, erythema, alopecia, irritation or necrosis around the site of administration (nasal cavity), locomotor activity, lacrimation, hyperthermia and hypothermia, etc. The weight of each animal was recorded once daily after first dose for a week and weekly once thereafter. Mean body weights and mean body weight gain was calculated for the corresponding intervals. The amount of feed consumed by each cage of animals was recorded once daily after first dose for a week and weekly once thereafter. Body temperature was recorded for rats and rabbits on day 0, 3 hours and 24 hours after each dose, and on the day of sacrifice.

## **Clinical Pathology Investigations:** Blood and urine samples were collected for clinical evaluations (hematology, coagulation parameters, acute phase proteins, serum chemistry and urinalysis) from all the groups. Samples for hematology and clinical biochemistry were collected on day 2 and 30 from main groups and on day 42 from the recovery groups. Urinalysis and Coagulation was performed in rat and rabbit on day 30 and 42. Acute phase proteins [Alpha 1-acid glycoprotein (α1-AGP) in rats and mice and C-reactive protein in rabbit] were evaluated in serum samples collected on days 0 (before dosing), 2, 30 and 42 using validated ELISA method (Life Diagnostics, USA).

Blood samples were collected from all animals under light isoflurane anesthesia (E-Z anaesthesia, Euthanex, USA) in rats and mice. Before blood collection, the animals were fasted overnight, allowing access to water *ad libitum*. Blood samples were drawn from the retro-orbital plexus using a micro-hematocrit heparinized glass capillary tube in rodents and from marginal ear vein in rabbits. Blood samples were centrifuged and the plasma was separated for clinical biochemistry and acute phase protein analyses. Hematology (Advia 2120, Siemens) parameters analyzed included erythrocyte count, haemoglobin, hematocrit, mean corpuscular volume, mean corpuscular haemoglobin, mean corpuscular haemoglobin concentration, platelet count, total leukocyte count, absolute and differential leukocyte count. Coagulation parameters (STA Compact®, Diagnostica Stago, France) such as Prothrombin time and Activated Partial Thromboplastin time were evaluated. Clinical biochemistry (Cobas C111 Analyser, Roche) parameters analyzed included glucose, urea, creatinine, total cholesterol, triglycerides, aspartate aminotransferase, alanine aminotransferase, alkaline phosphatase, Total bilirubin, sodium, potassium, chloride, total protein, albumin, globulin, and A/G ratio.

Urinalysis included volume, specific gravity, color, clarity, pH, red blood cells, white blood cells, bilirubin, ketone bodies, proteins, glucose and nitrite. Additionally, microscopic examination of urine was also carried out. Urine analysis was performed using Combur® Test Strips (Roche Diagnostics, Switzerland) with Urisys® 1800 (Roche, Switzerland).

## **Necropsy, Organ Weight and Histopathology:** Animals were euthanized by carbon dioxide asphyxiation (Smartbox, Euthanex, USA) for rats and mice and using thiopentone for rabbits, and necropsied. Organs as per WHO guideline which included nasal cavity, larynx, trachea, lungs, spleen, thymus and draining lymph nodes (cervical and mediastinal) were collected from all terminally sacrificed animals and macroscopic evaluation was recorded. Wet weights for organs such as brain, thymus, spleen, ovaries, uterus, heart, kidneys, testes, liver, adrenals, lungs and epididymides were recorded. Samples of organs were preserved in 10% neutral buffered formalin for microscopic examination. Testes and eyes were fixed in modified Davidson’s fixative for 24 hours and then transferred to NBF. For histopathology, tissues were processed, embedded in paraffin blocks, sectioned at 4 – 5 µ (Leica Biosystems, Germany) and stained with hematoxylin and eosin. Microscopic examination (Olympus, Japan) of slides was done by IBTP and DABT certified Veterinary Pathologist.

**Statistical Methods**: For ELISA and neutralizing antibody data analysis, statistical significance was assigned when *P* values were < 0.05 using Prism Version 9 (GraphPad). Data for body weight, feed consumption, organ weights and clinical pathology were analysed statistically using Statplus program. Following analysis for normality using Shapiro-Wilk W test, data for each group of animals were subjected to analysis of variance (ANOVA) and statistical significance was assigned and concluded at P value < 0.05. Values were presented as mean ± standard deviation (SD).

**Figure S1.** Mean body weight gain (%) for male (A) and female (B) Swiss Albino mice. G1 – Placebo (placebo); G2 – 5 x 10^9^ VP/animal; G3- 5 x 10^10^ VP/animal; G4 – 5 x 10^11^ VP/animal. The body weight gain in treated groups was comparable to placebo group.

**
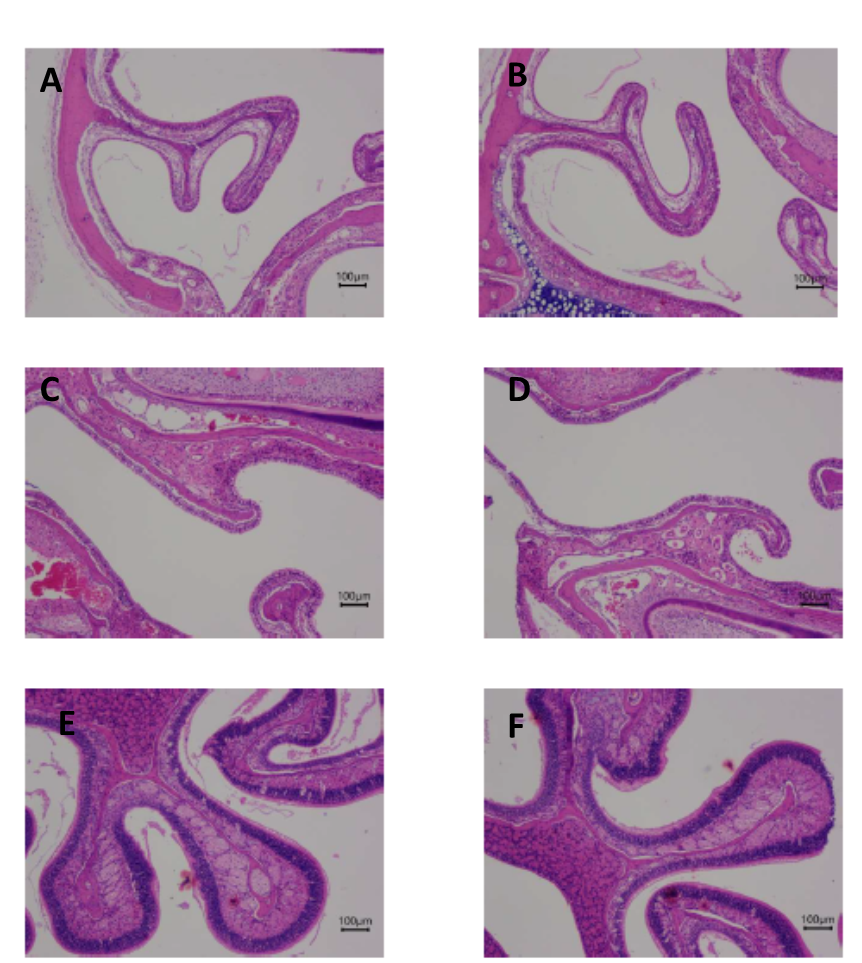
**

**Figure S2.** Light Microscopy images from Swiss Albino mice. Nasal Cavity, nasoturbinates (A - placebo, B - 5 x 10^11^ VP/animal), Nasal Cavity, maxilloturbinates (C- placebo, D - 5 x 10^11^ VP/animal), Nasal Cavity, ethmoturbinates (E- placebo, F - 5 x 10^11^ VP/animal). Nasal tissues were decalcified; blocks were prepared by embedding in paraffin and sectioned at approximate thickness of 4 - 5µ. Sections were taken on glass slides, stained with Hematoxylin & Eosin (H & E) and images were captured at 4X magnification. All tissues were within normal histological limits.


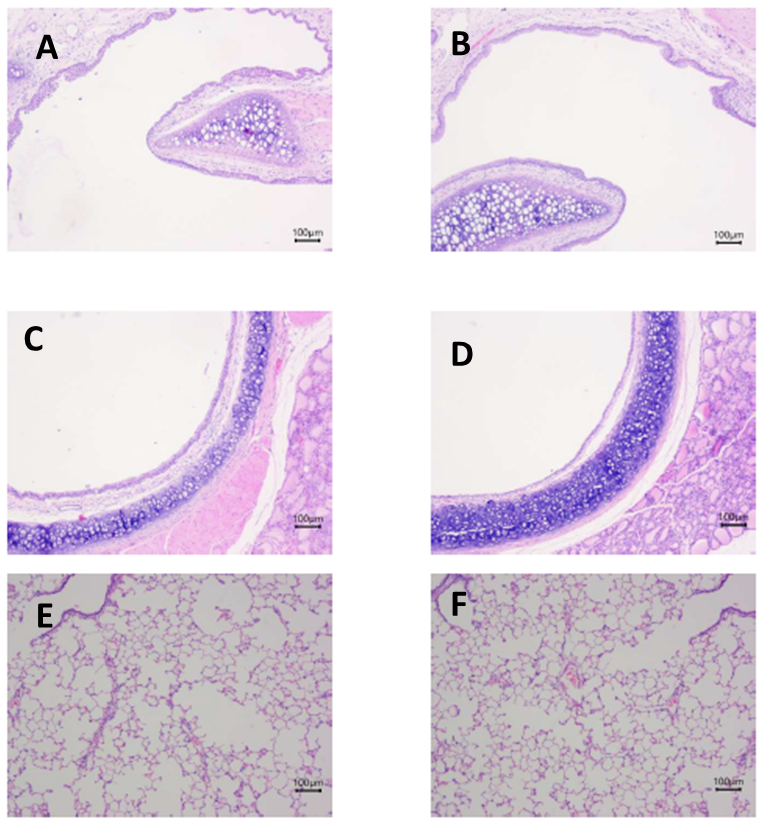


**Figure S3.** Light Microscopy images from Wistar rats. Larynx - (A - placebo, B - 5 x 10^11^ VP/animal), Trachea (C- placebo, D - 5 x 10^11^ VP/animal), Lungs (E - placebo, F - x 10^11^ VP/animal). Tissue blocks were prepared by embedding in paraffin and sectioned at approximate thickness of 4 - 5µ. Sections were taken on glass slides, stained with Hematoxylin & Eosin (H & E) and images were captured at 4X magnification. All tissues were within normal histological limits.

**Figure S4. S1-Specific IgG in BBV154 vaccinated mice**. Five to six-week-old BALB/c or Swiss Albino male and female mice (n=6/group) were immunized with 50 μl of placebo or BBV154 via intranasal route with 5x10^10^ VP/mouse. (A) S1-specific IgG in serum or BAL were evaluated in BALB/c mice at 2 weeks post-vaccination(B) S1-specific serum IgG was compared in BALB/c and Swiss Albino mice at 4 weeks post-vaccination (1x10^10^ VP/mouse). Data presented are from a single experiment, similar results were obtained in two independent experiments.

**Figure S5**.  **BBV154 induces strong systemic response (S1-IgM) in young versus aged Syrian hamsters.** Young (9–11-weeks-old), and old (28–36-weeks-old) male and female Syrian hamsters (n=10) were immunized intranasally with placebo or 1x10^11^ VP of candidate vaccine BBV154 in 100 μl. SARS-CoV-2 S1-specific IgM antibody responses in sera of immunized animals were evaluated four weeks post-priming. S1-specific IgM, in serum were measured by ELISA using individual sera (A). Significance was measured using nonparametric t-test: *, P < 0.05; **, P <0.01; ***, P < 0.001; ****, P < 0.0001.


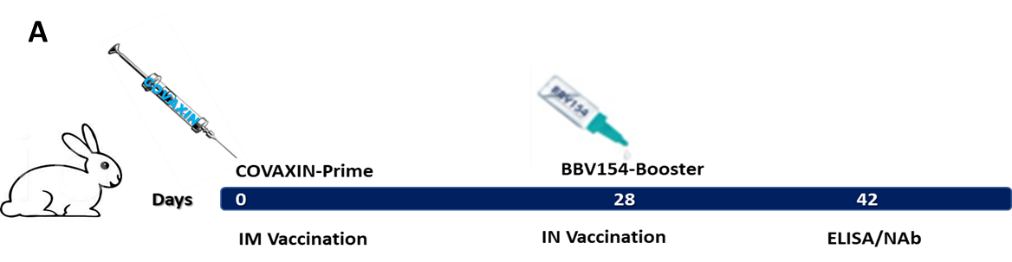

**Figure S6. Comparison of humoral immune responses induced by homologous or heterologous COVID-19 vaccines**. 10–12-week-old male and female New Zealand White Rabbits (n = 4) in two groups were immunized with heterologous (COVAXIN prime and BBV154 booster) or homologous (COVAXIN prime and COVAXIN booster) regimens on days 0 and 14 or 28 with 0.5ml [Human Single Dose (HSD)] of COVAXIN or BBV154 via IM or IN route respectively. (A). Two-weeks post-booster vaccination SARS-CoV-2 spike specific IgG (B) and IgA (C) were assessed by ELISA. Neutralizing activity of immune serum (E) against SARS-CoV-2 was performed by MNT50. Data presented are from a single experiment, similar results were obtained in two independent experiments.

**Figure S7**.  **Clinical batch of BBV154 vaccine elicited SARS-CoV-2 neutralizing antibodies that are comparable with COVID-19 convalescent samples.** 9–11-weeks-old male and female Syrian hamsters (n=10) were immunized intranasally with placebo or 1x10^11^ VP of candidate vaccine BBV154 in 100 μl. SARS-CoV-2 inhibition titers in sera of immunized animals were evaluated two weeks post-booster. The human convalescent serum (HCS) panel included specimens from participants with PCR-confirmed symptomatic and asymptomatic COVID-19 obtained at least at least 25 days after diagnosis (18 samples).

**Table S1.** Primers used in the current study

| **S. No.** | **Primer Name** | **Sequence (5’-3’)** | **Purpose** |
| --- | --- | --- | --- |
| 1. | Cassette F | TTTCCCGCGCTCTCTACAGG | For spike expression cassette |
| 2. | Cassette R | TCTTCCCCGCTGGAGCCGAA |  |
| 3. | Hexon F | GCGATGTTGATGTGTCCCAG | For hexon-based qPCR |
| 4. | Hexon R | TTCACCGCGGGGCCGTA |  |
| 5. | Hexon Probe | 6-FAM-ACAAGGAAGAGATGCTCCAGGTCATCGC-BHQ1 |  |
| 6. | Spike F | GCGATGTCTCCCAGGCAAT | For spike-based qPCR |
| 7. | Spike R | GGCGGATTCAACTTTTCCCAG |  |
| 8. | Spike Probe | 6-FAM-ATGAAGCCGGCATCAGCCAGGG-BHQ1 |  |

**Table S2. SUMMARY OF HEMATOLOGY FINDINGS – MALE WISTAR RATS ON DAY 31**

| **Group** | **RBC**  **x10^6^Cells/µL** | **Hb**  **g/dL** | **PCV**  **%** | **MCV**  **fL** | **MCH**  **pg** | **MCHC**  **g/dL** | **PLT**  **x10^3^cells/µL** | **WBC**  **x10^3^cells/µL** | **DC (x10^3^cells/µL)** | | | | |
| --- | --- | --- | --- | --- | --- | --- | --- | --- | --- | --- | --- | --- | --- |
|  |  |  |  |  |  |  |  |  | **NEUT** | **LYMPH** | **MONO** | **EOS** | **BASO** |
| PBS Placebo | 7.93±0.35 | 14.8±0.55 | 46.8±1.66 | 58.0±3.58 | 18.7±0.52 | 31.7±0.45 | 993.8±158.29 | 9.05±2.52 | 1.40±0.61 | 7.02±1.67 | 0.42±0.38 | 0.11±0.08 | 0.02±0.01 |
| 5 x 10^9^ VP | 8.67±0.45 | 15.98±0.73 | 50.57±2.39 | 58.32±0.38 | 18.45±0.26 | 31.63±0.33 | 1083.33±93.32 | 6.91±0.97 | 1.48±0.25 | 5.00±0.87 | 0.24±.07 | 0.10±0.03 | 0.02±0.01 |
| 5 x 10^10^ VP | 7.80±0.42 | 14.8±1.02 | 46.7±3.06 | 59.8±1.51 | 18.9±0.55 | 31.6±0.35 | 1206.2^*^±156.13 | 9.80±1.81 | 1.81±0.22 | 7.41±1.56 | 0.33±0.06 | 0.14±0.03 | 0.02±0.01 |
| 5 x 10^11^ VP | 8.24±0.20 | 15.12±0.32 | 47.14±1.06 | 57.20±0.45 | 18.36±0.33 | 32.10±0.44 | 984.20±49.84 | 8.26±0.82 | 1.47±0.21 | 6.36±0.57 | 0.21±0.03 | 0.11±0.08 | 0.02±0.01 |

* Significant at p ≤ 0.05 with Group 1

Abbreviations: RBC – red blood cell count, Hb-haemoglobin, PCV – Packed cell Volume (hematocrit), MCV - mean corpuscular volume, MCH - mean corpuscular haemoglobin, MCHC - mean corpuscular haemoglobin concentration, PLT -platelet count, WBC -total white blood cell (leukocyte) count, DC - differential cell count. NEUT – Neutrophils, LYMPH – lymphocytes, MONO – monocytes, EOS – eosinophils, BASO – basophils

**Table S3. SUMMARY OF CLINICAL BIOCHEMISTRY FINDINGS – MALE SWISS ALBINO MICE ON DAY 31**

| **GROUP** | **UREA**  **mmol /L** | **CREA**  **µmol/L** | **CHOL**  **mmol/L** | **TRIGL**  **mmol /L** | **GLU**  **mmol /L** | **ALP**  **U/L** | **ALT**  **U/L** | **AST**  **U/L** | **BIL**  **µmol/L** | **TP**  **g/L** | **ALB**  **g/L** | **GLB**  **g/L** | **A/G** |
| --- | --- | --- | --- | --- | --- | --- | --- | --- | --- | --- | --- | --- | --- |
| PBS Placebo | 10.78±1.59 | 16.58±3.76 | 3.35±0.59 | 1.70±0.52 | 8.12±1.19 | 74.63±16.92 | 36.92±6.85 | 90.15±39.46 | 0.28±0.36 | 56.93±1.66 | 30.36±3.46 | 26.58±3.27 | 1.18±0.27 |
| 5 x 10^9^ VP | 10.34±1.29 | 16.62±4.98 | 3.08±0.34 | 2.53±0.17 | 5.78±0.58 | 69.92±13.08 | 54.12±38.03 | 80.62±16.67 | 0.20±0.22 | 54.07±1.80 | 29.07±2.97 | 24.98±3.90 | 80.62±16.66 |
| 5 x 10^10^ VP | 9.46±0.97 | 23.15±6.96 | 3.76±0.45 | 1.30±0.31 | 8.89±2.02 | 74.30±23.47 | 40.40±20.91 | 83.10±12.09 | 0.35±0.50 | 61.30±7.22 | 28.67±2.22 | 32.65±6.44 | 0.92±0.16 |
| 5 x 10^11^ VP | 9.97±1.44 | 18.23±5.18 | 3.31±1.15 | 2.05±1.10 | 9.85±1.71 | 105.22±31.16 | 40.15±19.01 | 108.60±55.57 | 0.35±0.68 | 63.53±5.71 | 32.74±2.32 | 30.80±4.31 | 1.05±0.13 |

Abbreviations: GLU- Glucose, CREA- Creatinine, CHOL- Cholesterol, total, TRIGL- Triglycerides, AST- Aspartate aminotransferase, ALT-Alanine aminotransferase, ALP- Alkaline phosphatase, BIL- Bilirubin, Na- Sodium, K- Potassium, Cl- Chloride, TP- Total Protein, ALB -Albumin, GLB- Globulin, A/G - Albumin-globulin ratio
